# COVID‐19 and its effects on the digestive system and endoscopy practice

**DOI:** 10.1002/jgh3.12358

**Published:** 2020-05-17

**Authors:** Enrik John T. Aguila, Ian Homer Y. Cua, Joseph Erwin L. Dumagpi, Carlos Paolo D. Francisco, Nikko Theodore V. Raymundo, Marianne Linley L. Sy‐Janairo, Patricia Anne I. Cabral‐Prodigalidad, Marie Antoinette DC. Lontok

**Affiliations:** ^1^ Institute of Digestive and Liver Diseases St. Luke's Medical Center ‐ Global City Philippines

**Keywords:** COVID‐19, endoscopy, gastroenterology, liver, nutrition

## Abstract

The Coronavirus Disease 2019 (COVID‐19) is a respiratory illness caused by the severe acute respiratory syndrome coronavirus 2 (SARS‐CoV‐2) and has been classified as a pandemic by the World Health Organization in March 2020. Several studies have demonstrated that the gastrointestinal (GI) tract is also a potential route. As the pandemic is continuously evolving, and more data are made available, this article highlights the best evidence and practices regarding the effects of the SARS‐CoV‐2 virus relevant to GI practice. Published clinical studies have supported that SARS‐CoV‐2 affects the GI tract and the liver. The largest published dataset comprised of 4243 patients and showed a pooled prevalence of GI symptoms at 17.6%. GI symptoms varied and usually preceded pulmonary symptoms by 1–2 days. These include anorexia (26.8%), nausea and vomiting (10.2%), diarrhea (12.5%), and abdominal pain (9.2%). Incidence of liver injury ranges from 15 to 53%. Evidence shows that the severity of COVID‐19 infection is compounded by its effects on nutrition, most especially for the critically ill. As such, nutrition societies have recommended optimization of oral diets and oral nutritional supplements followed by early enteral nutrition if nutritional targets are not met, and parenteral nutrition in the distal end of the spectrum. In addition to possible fecal–oral transmission, GI endoscopy procedures, which are considered to be aerosol‐generating procedures, contribute to increased risk to GI health‐care professionals. Infection prevention measures and guidelines are essential in protecting both patients and personnel.

## Graphical abstract

With knowledge continuously evolving during the pandemic, this article highlights the current best evidence and practices regarding COVID‐19 that are relevant to the GI practice. The implications to the approach in the management of pre‐existing GI diseases, nutrition and practice of GI endoscopy are reviewed based on the guidelines of different GI societies worldwide.

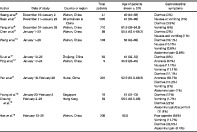

## Introduction

The Coronavirus Disease 2019 (COVID‐19) is a public health emergency caused by the severe acute respiratory syndrome coronavirus 2 (SARS‐CoV‐2) virus. This pneumonia pandemic began in China, which started last December 2019, and has spread to different countries. It has been reported that transmission of the virus is via human‐to‐human contact, droplet, and fomites.^1^ The incubation period for SARS‐CoV‐2 is around 2–14 days before symptoms develop; however, there have been reported cases of asymptomatic infection.^2^ Most patients with COVID‐19 develop fever along with respiratory symptoms, such as cough and dyspnea. Severe and critical conditions can develop into acute respiratory distress syndrome (ARDS), septic shock, and organ dysfunction, which can be fatal.^3^, ^4^ Although the virus spreads through respiratory droplets and secretions, the gastrointestinal (GI) tract can also be a potential route of viral transmission. Diarrhea, nausea and vomiting, and abdominal discomfort have been described in COVID‐19 patients.^2^, ^4^ Liver injury is also seen in severe cases.^5^

As the pandemic is continuously evolving and more data are being yielded daily, this article provides a consolidated update on the current information regarding the effects of SARS‐CoV‐2 virus on the GI tract, liver, nutrition, and the GI endoscopy practice. This article can also help in the development of policies and recommendations that can guide gastroenterologists, hepatologists, and other health‐care professionals during this time of pandemic.

## Methods

This review is based on different case reports, retrospective clinical studies, and society recommendations relating to the digestive system from articles published in the PubMed, EMBASE, and different international gastroenterology society websites. Extensive hand‐searching of the reference lists of papers, reports, and articles and cross‐referencing were done. The search terms used include “2019‐nCoV,” “SARS‐CoV‐2,” or “COVID‐19” combined with “gastrointestinal,” “clinical feature,” “digestive,” “liver,” “nutrition,” and “endoscopy.”

## Results and discussion

### *GI* *tract and* *COVID‐19*

#### *GI manifestations of* *COVID‐19*

Fever and respiratory problems remain the most common symptoms of COVID‐19. However, some patients were also reported to have atypical disease presentation. Pooled data from 11 articles showed that 2645 COVID‐19 patients from China, Hong Kong, and Singapore developed varied GI symptoms during the period of disease (Table 1).^3^, ^6^, ^7^, ^8^, ^9^, ^10^, ^11^, ^12^, ^13^, ^14^, ^15^ These GI symptoms include anorexia, nausea, vomiting, diarrhea, and abdominal pain. A recent meta‐analysis composed of 60 studies with 4243 patients from China, Singapore, South Korea, United Kingdom, and United States showed a pool prevalence of GI symptoms of 17.6%.^14^ Of these, anorexia was the most commonly reported symptom (26.8%), diarrhea in 12.5%, nausea and vomiting in 10.2%, and abdominal pain or discomfort in 9.2%. No significant subgroup difference between studies was found based on country of origin. GI symptoms were present in 17.1% patients with severe disease and 11.8% with nonsevere disease. These GI symptoms usually start early in the course of the disease and sometimes precede the onset of pulmonary symptoms.^3^, ^10^, ^11^, ^16^

**Table 1:** Gastrointestinal manifestations in patients diagnosed with COVID‐19

Author	Date of study	Country or region	Total patients	Age of patients (mean ± SD)	Gastrointestinal symptoms
Huang *et al*.^6^	December 16–January 2	Wuhan, China	41	49 (41–58)	Diarrhea (3%)
Guan *et al*.^7^	December 11–January 29	30 provinces in China	1099	47 (35–38)	Nausea or vomiting (5%) Diarrhea (3.8%)
Yang *et al*.^8^	December 24–January 26	Wuhan, China	710	51.9 (39–64.8)	Vomiting (5%)
Chen *et al*.^9^	January 1–20	Wuhan, China	99	55.5 (42.4–68.2)	Diarrhea (2%) Nausea and vomiting (1%)
Wang *et al*.^10^	January 1–28	Wuhan, China	138	56 (42–68)	Diarrhea (10.1%) Nausea (10.1%) Vomiting (3.6%) Abdominal pain (3.6%)
Xu *et al*.^11^	January 10–26	Zhejiang, China	62	41 (32–52)	Diarrhea (8%)
Ping *et al*.^12^	January 17–24	Wuhan, China	9	35.8 (28–45)	Anorexia (67%) Nausea (11.1%) Vomiting (11.1%) Diarrhea (11.1%)
Pan *et al*.^3^	January 18–February 28	Hubei, China	204	52.9 (51.3–68.9)	Anorexia (39.7%) Diarrhea (17.2%) Vomiting (2%) Abdominal pain (1%)
Young *et al*.^13^	January 23–February 3	Singapore	18	47 (31–73)	Diarrhea (17%)
Cheung *et al*.^14^	February 2–29	Hong Kong	59	58.5 (43.5–68)	Vomiting (1.7%) Diarrhea (22%) Abdominal pain/discomfort (11.9%)
Han *et al*.^15^	February 13–29	Wuhan, China	206	62.5	Poor appetite (34%) Vomiting (11.7%) Diarrhea (32.5%) Abdominal pain (4.4%)

Table 1 shows the findings of the 11 studies included in the pooled analysis of COVID‐19 patients related to GI symptoms. Anorexia or poor appetite was most common (34–67%), followed by diarrhea (2–17.2%), vomiting (1–11.7%), and nausea (1–11.1%). Diarrhea is characterized by loose stools occurring at least thrice daily and is not voluminous or severe and usually lasts up to 14 days.^3^, ^14^ A case–control study by Han *et al*. estimated that around 20% of patients had diarrhea as the presenting symptom. GI symptoms usually preceded pulmonary symptoms by at least 1–2 days.^15^ A recent case report also documented SARS‐CoV‐2 GI infection causing hemorrhagic colitis.^17^

It is estimated that about half of admitted patients had at least one GI symptom, with these symptoms becoming more pronounced as the severity of the disease progressed.^3^ Patients with GI symptoms were also found to have longer time from symptom onset to admission. They were more likely to develop liver injury and receive antibiotic treatment.^3^, ^15^

#### *Proposed pathophysiologic mechanisms for GI manifestations of* *COVID‐19*

The pathogen responsible for COVID‐19 is officially named severe acute respiratory syndrome coronavirus 2. It is a novel enveloped RNA betacoronavirus, which is phylogenetically similar to SARS‐CoV and closely related to other betacoronaviruses of bat origin, indicating that these animals are the likely reservoir hosts for this emerging viral pathogen.^18^ The angiotensin‐converting enzyme 2 (ACE2) receptor is the receptor used by SARS‐CoV‐2 to allow its entry into the target cell and facilitate replication.^19^, ^20^ These receptors have been found to be expressed in the enterocytes in the ileum and colon. ACE2 staining of biopsy specimens showed that the positive areas were mainly distributed in the cytoplasm of gastric and intestinal epithelial cells and the cilia of glandular epithelial cells but were rarely observed in esophageal squamous epithelial cells. Viral nucleocapsid protein was detected in the cytoplasm of gastric, duodenal, and rectal glandular epithelial cell but not in the esophageal epithelium.^18^ In addition, the SARS‐CoV‐2 virus is also believed to disrupt the normal intestinal flora, leading to GI symptoms, especially diarrhea.^3^

Binding of the virus to the ACE2 receptors found in liver and bile duct epithelial cells causes liver injury. This results in elevation of aminotransferases, decrease in albumin, and prolongation of prothrombin time. The SARS‐CoV‐2 virus affects the digestive system directly by causing tissue damage and indirectly by producing an inflammatory reaction resulting in changes in the intestinal flora, leading to symptoms of diarrhea.^3^ Furthermore, the SARS‐CoV‐2 virus has been detected in the stools of infected patients and can remain positive for the virus even if it has been cleared in the respiratory tract.^2^, ^21^

#### *Fecal tests for* *SARS‐CoV‐2 infection*

The presence of SARS‐CoV‐2 RNA in the stool was first documented by Holshue *et al*., when the first confirmed case in the United States, a 35 year‐old man with a recent travel to Wuhan, China, developed diarrhea on the sixth day of illness.^16^ Recent studies estimate the proportion of patients whose stool samples tested positive for the SARS‐CoV‐2 RNA to be 35.7–54.5% of all confirmed cases (Table 2). Zhang *et al*. reported high accuracy in detecting nucleic acids in stool samples. However, this accuracy is not directly related to disease activity and the presence of GI symptoms.^19^ Cheung *et al*. were able to estimate the median viral load at 4.7 log_10_ copies per mL, with higher stool viral load noted among patients with diarrhea.^14^

**Table 2:** Fecal SARS‐CoV‐2 RT‐PCR test in patients with COVID‐19

Author	Total patients	Patients with positive fecal RT‐PCR
Cheung *et al*.^14^	59	9 (15.3%)
Han *et al*.^15^	22	12 (54.5%)
Xiao *et al*.^20^	73	39 (53.4%)
Zhang *et al*.^18^	14	5 (35.7%)

A subgroup analysis conducted by Cheung *et al*. also showed detection of the viral RNA in stool. In their analysis, viral RNA was detected in both stool and respiratory specimen in 68 of 138 patients, with a pooled prevalence of 48.1%. The subgroup with a positive stool viral RNA but negative respiratory samples had a higher pooled prevalence (70.3%) than the subgroup with positive viral RNA in both respiratory and stool specimens. Patients on steroids were also noted to have a longer stool viral clearance rate compared to those who were not on steroid use.^14^

The presence of SARS‐CoV‐2 viral nucleic acids in stool has raised suspicion that fecal excretion, environmental contamination, and fomites might contribute to viral transmission. The virus has recently been found to be stable and viable for up to 72 h on plastic, stainless steel, copper, and cardboard surfaces.^18^

#### *Management of GI Manifestations of* *COVID‐19*

Recent studies, guidelines, and treatment protocols regarding COVID‐19 patients with GI symptoms have not elaborated much on the management of these symptoms. A review by Tian *et al*. showed that the National Health Commission of the People's Republic of China advocated symptomatic treatment of these symptoms. Adequate hydration was the cornerstone of treatment to prevent electrolyte imbalance. Drugs such as dioctahedral montmorillonite powder and loperamide were used to alleviate diarrhea. Probiotics were given to mitigate intestinal dysbiosis and possibly reduce bacterial translocation and secondary infection. Antispasmodics were given to help relieve abdominal pain.^22^

### *Liver and* *COVID‐19*

COVID‐19 is mainly a respiratory illness, but liver injury may occur during the course of the disease. It is more commonly seen among patients with severe cases of COVID‐19.^23^, ^24^ The mechanism of liver injury is unknown but is attributed to the abundance of ACE2 receptor in the endothelial cells of the liver and bile duct cell.^24^, ^25^ ACE2 receptors are used by the virus for cell entry, and its expression is noted to be higher in bile duct cells than liver cells. Because of this, liver injury is postulated to be secondary to damage in the bile duct cells. Bile duct cells are known to play an important role in hepatocyte regeneration and liver immune response. Among patients who are critically ill, immune‐mediated inflammation, such as cytokine storm and pneumonia‐associated hypoxia, might also contribute to liver injury. Whether the elevation of liver enzymes is due to direct liver injury or immune‐mediated liver injury has not been resolved. Furthermore, drug‐induced liver injury may occur among these patients as different medications, such as antibiotics, antivirals, or steroids, are used, potentially causing or aggravating liver injury.

The incidence of liver injury ranges from 15‐53%.^23^ Laboratory findings include elevated alanine aminotransferase (ALT) and aspartate aminotransferase (AST), slight increase in bilirubin level, elevated gamma‐glutamyl transferase, elevated alkaline phosphatase, low albumin level, and prolonged prothrombin time.^23^, ^24^, ^26^ A low albumin level is considered a marker of COVID‐19 disease severity.^26^

**Figure 1:**
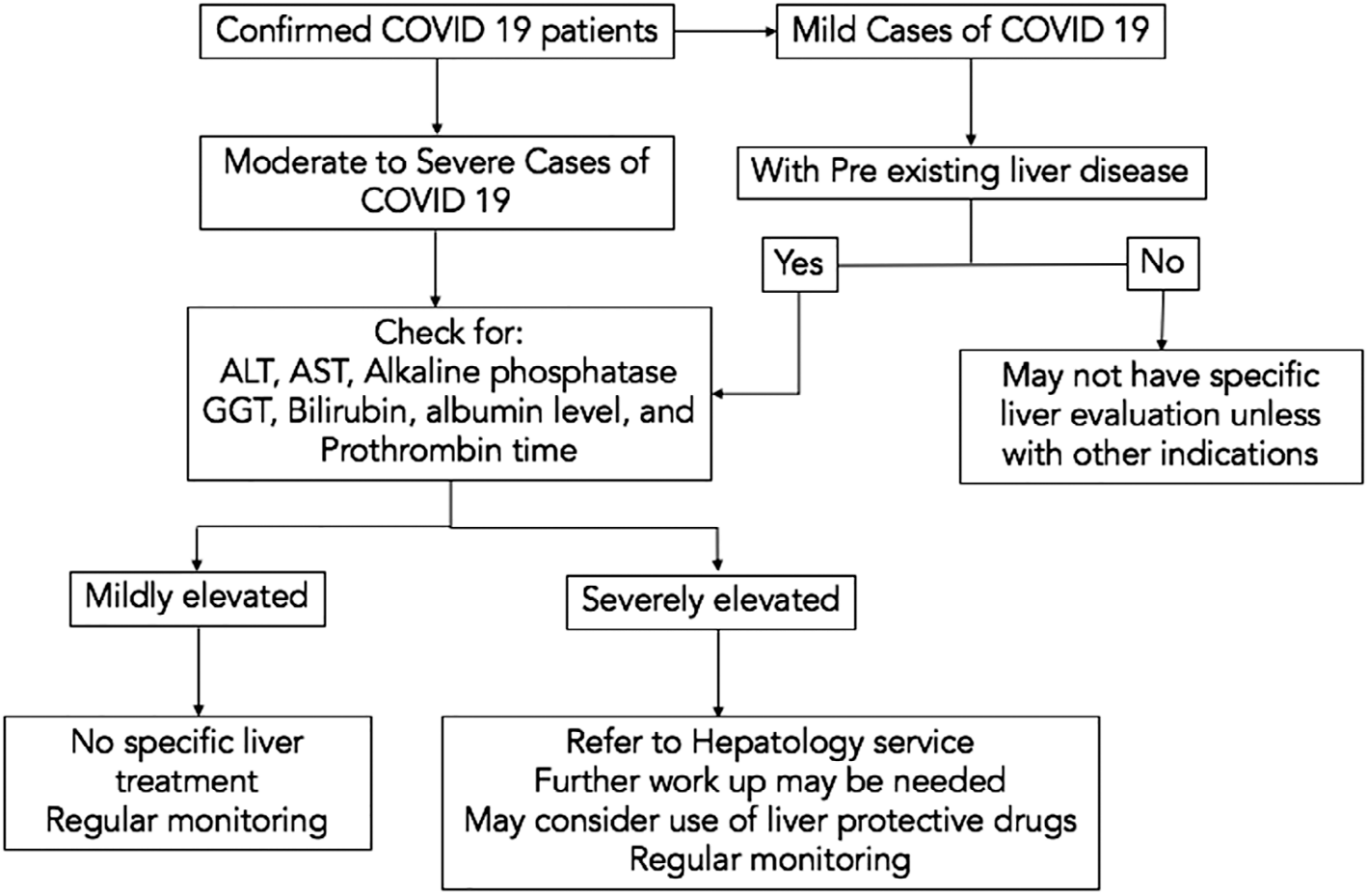
Algorithm for the evaluation and management of liver abnormalities among COVID‐19 patients.

Larger numbers of patients with liver injury have severe cases of COVID‐19. In the majority of patients, liver injury is transient and does not require specific medications.^23^, ^24^ Regular monitoring of liver enzymes is recommended. Screening for hepatitis B and hepatitis C should also be performed.^23^ Figure 1 shows an algorithm for the evaluation and management of liver abnormalities among COVID‐19 patients. Patients with underlying liver disease are considered to be more vulnerable to severe disease, but there is no strong evidence of risks of reactivation of viral hepatitis or decompensation among patients with chronic liver disease. ^23^ These patients warrant close monitoring.

Currently, several COVID‐19‐specific therapies are being evaluated. Concerns for drug–drug interaction and safety exist. Therapeutic agents used to manage symptomatic COVID‐19 that can be hepatotoxic include HMG‐CoA reductase inhibitors (statins), remdesivir, and tocilizumab. Patients with elevated liver enzymes should still be considered for investigational medications, although an ALT or AST of more than five times the upper limit normal is an exclusion criteria for some investigational medications.^23^ Monitoring of liver chemistries is recommended regardless of the baseline value. For analgesics and antipyretics, the preferred medication is acetaminophen of 2 g/day. Regular review for drug–drug interactions is advised.

### *Inflammatory bowel disease and* *COVID‐19*

Inflammatory bowel diseases (IBD), such as Crohn's disease and ulcerative colitis, are chronic, autoimmune, and immune‐mediated inflammatory diseases, whose pathogenesis includes genetic susceptibility and environmental factors.^27^ The risk of having an opportunistic infection is increased in patients with IBD, especially for patients who are older than 50 years and are receiving immunosuppressive therapy.^28^ The number of ACE2 receptors is significantly higher in the inflamed intestinal lining of IBD patients. Given the state of the impaired immune system, the type of treatment of IBD, and the increased number of ACE2 receptors in their intestinal linings, these patients might be assumed to be at higher risk of acquiring the SARS‐CoV‐2 virus. Nevertheless, studies show that the susceptibility to COVID‐19 infection of IBD patients is similar to the normal population.^27^, ^28^, ^29^

In this era of the COVID‐19 pandemic, it is still possible to maintain a high quality of care for IBD patients. The International Organization for the Study of Inflammatory Bowel Diseases (IOBD), Crohn's and Colitis UK and Crohn's and Colitis Foundation recommended not to suspend ongoing medical treatment for IBD. Treatment with thiopurines have been associated with an increased risk of viral infection. However, the IOBD recommends continuing this drug as it take months to be eradicated from the system, and stopping it for a short term will not be beneficial.^30^ The use of mesalamine should be continued because it does not increase the risk of infection.^31^ There has been no strict evidence regarding the use of steroids in COVID‐19 patients. The use of low‐dose steroids on a short‐term basis is not associated with worse prognosis in severe COVID‐19 infection and therefore can be used to treat IBD flares.^32^ Vedolizumab can be continued because of its specificity for the intestine.^31^ Ustekinumab can also be continued; however, starting treatment with this drug is not encouraged because of the need of an infusion center.

Patients who are receiving immunosuppressants must be monitored carefully for any signs and symptoms of COVID‐19 infection.^27^ It is recommended that initiation of immunosuppressive drugs and biologics be delayed if the patient is asymptomatic.^28^, ^32^ In cases where treatment must be started, screening of hepatitis B virus, hepatitis C virus, HIV, varicella zoster virus (in patients with no history of infection), and tuberculosis is advised. Currently, there is no guideline for screening prior to initiation of therapy, but an article by Zingone has recommended including testing for SARS‐CoV‐2 during this time of pandemic.^28^

Surgery and endoscopy are part of the management options for IBD patients. Elective surgery and endoscopy must be postponed. IBD‐related indications for endoscopy during this pandemic are the following: for confirmation of a new diagnosis in moderate to severe IBD, for evaluation of severe acute flare‐ups in ulcerative colitis, for evaluation of partial or subacute bowel obstruction, and in known IBD patients with primary sclerosing cholangitis who develop cholangitis.^33^ For cases where surgery and endoscopic procedures on IBD patients are contemplated, screening for COVID‐19 infection is recommended.^31^, ^32^

### *Nutrition support in the* *COVID‐19 treatment*

Evidence shows that the severity of COVID‐19 infection is compounded by its effects on nutrition, especially for the critically ill where loss of skeletal muscle mass and function is apparent.^34^ Prolonged hospital stays, particularly ICU days, is a well‐documented cause of malnutrition because of reduced mobility and catabolic changes, particularly in the skeletal muscle, as well as reduced food intake.^35^ Furthermore, older age, chronic diseases, and their clustering in polymorbid individuals confer higher risk of malnutrition and worse outcomes. Thus, nutrition plays an important role in the overall management of patients with COVID‐19 disease.

The American Society for Parenteral and Enteral Nutrition (ASPEN), in cooperation with the Society of Critical Care Medicine, has formulated a guideline on the *Nutrition Therapy in the Patient with COVID‐19 Disease Requiring ICU care*. The European Society for Clinical Nutrition and Metabolism (ESPEN) also published their *Expert Statements and Practical Guidance for Nutritional Management of Individuals with the COVID‐19 Infection*. In general, these guidelines have recommended optimization of oral diets and oral nutritional supplements whenever possible to meet patient's needs. Enteral nutrition is recommended if the nutritional requirements cannot be met orally. Parenteral nutrition should be considered if enteral nutrition is still unable to reach targets. Regular physical activity should be also emphasized, whenever feasible.^35^

Vitamins and minerals supplementation potentially reduces the disease's negative impact by maximizing general anti‐infective nutritional defense. ^35^ However, there is no established evidence that routine, empirical use of a supratherapeutic amount of micronutrients may improve clinical outcomes of COVID‐19. Supplementation of vitamins and minerals (e.g. vitamins A, B, C, D, and E; omega‐3 polyunsaturated fatty acids; selenium; zinc; and iron) is only recommended for malnourished patients who are at risk of or have COVID‐19.

Placement of enteral access, such as a nasogastric tube, is an aerosol‐generating procedure (AGP) as it may provoke coughing.^34^ Proper personal protective equipment (PPE) is recommended for use based on the CDC guidelines.

For the critically ill admitted to the ICU, early enteral nutrition is advised to start at hypocaloric prescription in trophic rates. Continuous feeding is recommended to reduce incidence of diarrhea. Prokinetics are advised to enhance motility if there is GI intolerance, such as vomiting or high gastric residual volumes.^35^ Early parenteral nutrition should be considered if GI symptoms are present, such as unexplained abdominal pain, nausea, vomiting, diarrhea, and significant abdominal distention. The threshold for switching to parenteral nutrition in patients with COVID‐19 disease may need to be lowered so as to minimize the risk of ischemic bowel and reduce additional unnecessary exposure of health‐care professionals.^34^ Nasojejunal feeding is also an option for patients with GI intolerance despite use of prokinetics. The enteral formula should be of the standard high protein polymeric isosmotic type. As the patient and the GI dysfunction improves, fiber should be added for the nonnutritional benefits to the gut microbiota. Studies have also suggested the benefit of fish oil‐containing formulas in immune modulation and the clearing of viral infections.

As COVID‐19 may lead to ARDS necessitating mechanical ventilation, some patients may develop refractory hypoxemia, and practices have shown prone positioning to be beneficial in oxygenation.^34^, ^35^ Nutrition societies recommend early enteral nutrition for these patients. It is recommended to keep the head of the bed elevated or in a reverse Trendelenburg position to at least 10–25° to decrease risk of aspiration.^34^ Feeding is also started at a hypocaloric prescription, which is slowly progressed if there are no signs of GI intolerance. This is to reduce the risk of aspiration while optimizing adequate provision of nutrition. A restrictive fluid management strategy is also recommended to avoid fluid overload and respiratory failure.^36^

### *Digestive endoscopy service in the* *COVID‐19 pandemic*

Endoscopy recommendations have been changing rapidly. A study by Filho *et al*. has reviewed different guidelines and statements from international gastroenterology societies.^37^ Twenty‐one societies elaborated specific recommendations for endoscopy during the COVID‐10 pandemic. Table 3 shows the percentage statistics in relation to the recommendations given by the GI societies.

**Table 3:** Review of different gastroenterology society recommendations (Filho *et al*., 2020)

Recommendations	Percentage
Use of PPE during the examination	100%
Temporarily postpone nonurgent procedures	95%
Stratify patients for risk of COVID‐19 before the examination (questionnaire of symptoms and/or patient's body temperature)	86%
Reduce the number of people who accompany patients	38%
Stimulate self‐surveillance of signs/symptoms by HCW	33%
Contact patients 14 days after examination to check symptoms	19%

During pandemics, every patient should be considered and assumed a potential COVID‐19 carrier; hence, the need for individual risk stratification. All patients should be screened and stratified based on the following criteria:

1. Presence of at least one symptom suspicious for COVID‐19, which includes fever (>37.5°C or 99.5°F), diarrhea, nausea, vomiting, abdominal discomfort, dry cough, sore throat, runny nose, or nasal congestion
2. Recent travel history to any high‐risk area or countries with level 3 travel health notice from Centers for Disease Control and Prevention (CDC) within the past 2 weeks
3. Close contact or possible exposure with a suspicious or confirmed case of COVID‐19

Patients are screened and classified as low, intermediate, and high risk based on the American Society for Gastrointestinal Endoscopy (ASGE) classification (Table 4). In an emergency setting, all patients must be considered high risk if adequate history cannot be assessed.^38^

**Table 4:** Classification of potential SARS‐CoV‐2 infection risk in patients undergoing endoscopy

Low Risk	No symptom No contact with SARS‐CoV‐2‐positive person No stay in high‐risk area during the previous 14 days
Intermediate Risk	Presence of symptoms with No medical history of contact with SARS‐CoV‐2‐positive person No stay in high‐risk area during the previous 14 days No symptoms but with Contact with SARS‐CoV‐2‐positive person Stay in high‐risk area during the previous 14 days
High Risk	At least 1 symptom +1 of the following: Contact with SARS‐CoV‐2‐positive person Stay in high‐risk area during the previous 14 days

Health‐care professionals are at increased risk for COVID‐19 because of the potential of airborne transmission in upper GI endoscopy and the growing evidence of fecal‐oral transmission. AGPs such as upper GI endoscopy are at high risk for COVID‐19 transmission.^38^, ^39^ Fecal contamination via inhalation, conjunctival splash contact, or direct contact with feces during colonoscopy are potential modes of transmission.^40^

**Figure 2:**
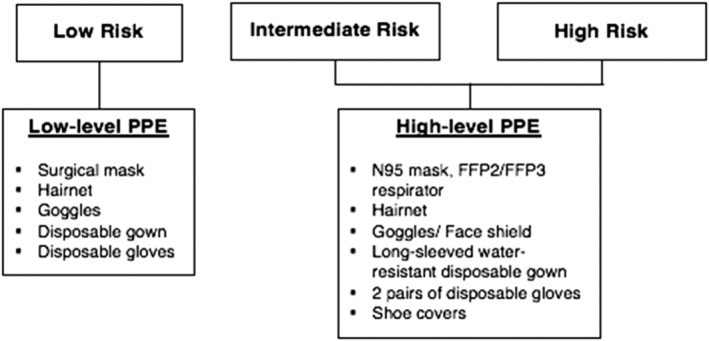
Level of personal protective equipment (PPE).

To prevent transmission of COVID‐19 within the endoscopy unit, infection control measures should be established. These measures are aimed to reduce the risk of the spread of infection from possible COVID‐19 patients, reduce the risk of cross‐infection, and preserve the use of PPE. Appropriate and adequate PPE for the staff is essential to limit the spread of the virus. The set and type of PPE used for the procedure should be modified based on the risk stratification of each patient, as shown in Figure 2.^38^

In order to minimize the exposure of patients and health‐care professionals to the virus, endoscopic procedures should strictly be limited to emergency or essential GI procedures (Table 5). All elective and nonurgent procedures should be deferred and rescheduled until the outbreak is resolved.^38^, ^39^, ^40^, ^41^, ^42^ However, the British Society of Gastroenterology (BSG) recommends that some procedures may be considered urgent and need further discussion on a case‐by‐case basis at a consultant level. These procedures include but are not limited to cancer biopsy/staging referrals and planned endoscopic mucosal resection (EMR) or endoscopic submucosal dissection (ESD) for complex/high‐risk lesions.^43^

**Table 5:** Category of endoscopic procedures

Emergent	Urgent	Elective
Ascending/acute cholangitis Foreign body retrieval Gastrointestinal obstruction Life‐threatening GI bleeding	Cancer staging Stable GI bleeding Tumor biopsy Palliative procedures (stenting, neurolysis) Planned EMR/ESD for complex/high‐risk lesions	Biliary stent removal Clinical trials Colorectal cancer screening Percutaneous endoscopic gastrostomy tube insertion Post‐polypectomy surveillance Surveillance/follow‐up endoscopy (excluding high risk neoplasia)

The number of endoscopy personnel and operational endoscopy rooms should be limited. Endoscopy work teams should be composed of a consultant endoscopist, consultant anesthesiologist, and a highly trained endoscopy nurse to limit procedure time, avoid prolonged exposure, and preserve supplies of PPE. The involvement of trainees, including GI fellows, should be limited.^40^, ^42^ Social distancing of at least 3–6 feet between persons should be observed. The ASGE suggests that all procedures should be performed in a negative‐pressure room.^38^ Each staff member involved should follow the standard CDC infection control precautions, which include hand washing with soap and water or rubbing with alcohol‐based solution.

After the endoscopic procedure, different societies have recommended that all used endoscopes must undergo standardized reprocessing and disinfection. In high‐risk or confirmed COVID‐19 cases, double disinfection is mandatory. The disinfectant to be used must be bactericidal, mycobactericidal, fungicidal, and virucidal against enveloped and nonenveloped viruses.^41^ All used accessories should be disposed of immediately in the appropriate medical infectious waste bin and must not be reused.^40^ The disinfection process of each endoscopy suite and the recovery room should include cleaning of all surfaces to remove clinical soil and biofilms, followed by thorough disinfection after every case. Wall‐to‐wall disinfection, UV irradiation, and ozone treatment for cleaning of air and surfaces are advised, as all surfaces should be considered contaminated. A delay or interval of 1 h between procedures is recommended to allow thorough disinfection as small particles remain airborne for some period of time. Chlorine‐containing (1000 ppm active chlorine) detergent or 1:100 dilution of household bleach and water is recommended for floor cleaning daily.^38^, ^40^, ^42^

## Discussion

The current COVID‐19 pandemic has a great impact on gastroenterology care as growing knowledge has supported that the SARS‐CoV‐2 virus affects the GI tract and the liver. The postulated strong affinity of the virus to ACE2 receptors expressed on intestinal cells and hepatocytes allows its entry into the target cell.^19^ The normal intestinal flora is disrupted, leading to GI symptoms such as diarrhea.^3^ Immune‐mediated inflammation such as cytokine storm contributes to the liver injury.^23^

Epidemiological studies showed that COVID‐19 patients develop varied GI symptoms during the period of the disease, which include anorexia, nausea, vomiting, diarrhea, and abdominal pain. The largest published meta‐analysis, which is comprised of 60 studies, showed a pooled prevalence of GI symptoms at 17.6%.^14^ It is important to note, however, that most of these studies (*n* = 53) came from China, with the rest from South Korea, Singapore, Vietnam, United States, and United Kingdom. Studies with large sample size in other geographic locations could lead to a more precise estimate of the prevalence of GI manifestations. The majority of studies only reported GI symptoms on the day of admission but not throughout the course of disease; therefore, further data are recommended to be able to determine the true prevalence of GI symptoms in this pandemic. GI manifestations may be the only initial symptoms in some COVID‐19 patients; hence, these should not be overlooked, and health‐care professionals should always exercise a high index of suspicion.

Evidence of the potential fecal–oral transmission should also be further investigated with the detection of viral RNA in the stool. Existing studies showed that the proportion of patients with a positive fecal test for SARS‐CoV‐2 nucleic acid ranged from 35.7 to 54.5% of all confirmed cases.^14^, ^15^, ^18^, ^20^ Studies have also shown that some patients have longer stool viral clearance rates.^14^ More data are required to formulate guidelines for the protection of patients in terms of this possible transmission route.

COVID‐19 has also affected clinical practice in several ways. Gastroenterologists attend to their outpatients through teleconferencing, especially in the follow up of patients with preexisting GI diseases. Patients with chronic liver disease or IBD are advised to continue their medications with careful monitoring.^23^, ^27^ Patients in the ICU are presented with different nutrition dilemmas, with GI intolerance and nutrition routes posed as the larger problems. Nutrition societies have recommended early enteral nutrition if targets are not met and the use of prokinetics if there are signs of GI intolerance. The threshold for switching to parenteral nutrition in patients with COVID‐19 disease is also lowered.^34^

GI endoscopy procedures that are considered to be AGPs contribute to increased risk to GI health‐care professionals.^38^, ^39^ Different endoscopic guidelines have continuously evolved to be able to address the prevention of further spread of the virus.^37^ Endoscopy units have strictly limited their procedures and cater only to emergency cases. Gradual resumption of endoscopy services should be carefully planned in the context of the growing pandemic.

In conclusion, digestive symptoms are common in patients with COVID‐19. A high index of suspicion in at‐risk patients presenting with these symptoms may help with the early detection and treatment of the disease. Nutrition support should be emphasized to improve clinical outcomes. GI endoscopic procedures should follow strict infection control protocols and should strictly be limited to emergency procedures to minimize viral exposure of patients and health‐care professionals.
